# Evaluating the Impact of Putative Metformin Targets on Cancer Outcomes: A Drug‐Target Mendelian Randomization Study

**DOI:** 10.1111/dom.70598

**Published:** 2026-02-27

**Authors:** Xingyu Shen, Shan Luo, Jie Zheng, Celine Sze Ling Chui, Ian Chi Kei Wong, Eric Yuk Fai Wan, Catherine Mary Schooling, Shiu Lun Au Yeung

**Affiliations:** ^1^ School of Public Health, Li Ka Shing Faculty of Medicine The University of Hong Kong Hong Kong (Special Administrative Region) China; ^2^ Department of Family Medicine and Primary Care, Li Ka Shing Faculty of Medicine The University of Hong Kong Hong Kong (Special Administrative Region) China; ^3^ Department of Endocrine and Metabolic Diseases Shanghai Institute of Endocrine and Metabolic Diseases, Ruijin Hospital, Shanghai Jiao Tong University School of Medicine Shanghai China; ^4^ Shanghai National Clinical Research Center for Metabolic Diseases, Key Laboratory for Endocrine and Metabolic Diseases of the National Health Commission of the PR China, Shanghai Key Laboratory for Endocrine Tumor, Shanghai Digital Medicine Innovation Center, Ruijin Hospital. Shanghai Jiao Tong University School of Medicine Shanghai China; ^5^ MRC Integrative Epidemiology Unit (IEU), Bristol Medical School, University of Bristol Bristol UK; ^6^ School of Nursing, Li Ka Shing Faculty of Medicine The University of Hong Kong Hong Kong (Special Administrative Region) China; ^7^ Centre for Safe Medication Practice and Research, Department of Pharmacology and Pharmacy, Li Ka Shing Faculty of Medicine The University of Hong Kong Hong Kong (Special Administrative Region) China; ^8^ Aston Pharmacy School Aston University Birmingham UK; ^9^ School of Pharmacy, Medical Sciences Division Macau University of Science and Technology Macau (Special Administrative Region) China; ^10^ Advanced Data Analytics for Medical Science Limited Hong Kong (Special Administrative Region) China; ^11^ The Institute of Cardiovascular Science and Medicine, Li Ka Shing Faculty of Medicine The University of Hong Kong Hong Kong (Special Administrative Region) China; ^12^ Comprehensive Primary Healthcare Collaboratory, Li Ka Shing Faculty of Medicine The University of Hong Kong Hong Kong (Special Administrative Region) China; ^13^ Graduate School of Public Health and Health Policy City University of New York New York City New York USA

**Keywords:** cancer, genome‐wide association study (GWAS), Mendelian randomization, metformin, reposition

## Abstract

**Aims:**

Observational studies show metformin use associated with lower cancer risk, although experimental evidence is inconsistent. To provide genetic validation for repositioning of metformin in cancer prevention, we assessed genetically proxied effects of putative metformin targets on cancer outcomes using a drug‐target Mendelian randomization (MR) design.

**Materials and Methods:**

We identified genetic proxies of 11 metformin targets (*PRKAA1, PRKAA2, PRKAB1, PRKAB2, PRKAG1, PRKAG2, PRKAG3, ETFDH, GPD1, SLC47A1* and *ACACB*) based on their associations with tissue‐specific gene expression, overall/sex‐specific HbA1c and type 2 diabetes. We then evaluated genetically proxied effects of these targets on five major cancers using MR. We also employed a conventional MR design to assess the relationship of HbA1c with cancer using the inverse variance method, with sensitivity analyses. Associations were corrected for multiple comparisons using false discovery rates.

**Results:**

We identified two genetic proxies of putative metformin targets (*PRKAG1* and *GPD1*) as valid instrumental variables (F statistics > 10). *PRKAG1* was associated with a reduced risk of colorectal cancer (OR: 0.74 per mmol/mol reduction in overall HbA1c, 95% CI: 0.63–0.87; *p* = 0.001), with consistent findings in sex‐specific analysis. This effect was unlikely mediated by HbA1c reduction, as indicated by conventional MR analyses (OR: 1.01 per mmol/mol, 95% CI: 0.99–1.02). No significant association was observed for *GPD1* (OR: 1.00, 95% CI: 0.74–1.36; *p* = 0.98).

**Conclusions:**

Metformin may prevent colorectal cancer via the AMPKγ1 (*PRKAG1*) target based on genetic evidence, supporting the evaluation of metformin use in colorectal cancer prevention using randomised controlled trials.

## 1 Introduction

Cancer remains a key contributor to the global disease burden, with 20 million new cases and 9.7 million deaths reported in 2022 by the World Health Organization [1]. Driven by ageing populations and lifestyle factors (e.g., sedentary behaviour, sleep deprivation and alcohol use), incidence is projected to rise by 77% by 2050, exceeding 35 million new cases [1]. Given the substantial burden, repurposing existing medications for cancer prevention offers a cost‐effective alternative to novel drug development, which faces high costs and uncertain long‐term safety [2].

Metformin, a lipophilic biguanide, a first‐line treatment for type 2 diabetes (T2D), has been linked to reduced cancer risk in pharmacoepidemiologic studies [3], including breast [4], colorectal [5] and prostate cancer [6]. However, these findings may be influenced by confounding by indication and immortal time bias [7, 8], although the extent of such bias remains debated [9]. Nevertheless, a target trial using electronic health records [10] and a meta‐analysis of randomised controlled trials (RCTs) [11, 12] showed no effect of metformin on lowering cancer risk, highlighting the need for more robust designs to evaluate metformin's potential anti‐cancer effects [13].

Mendelian randomization design is a genetic approach, which utilises genetic variants randomly inherited at conception, and hence is more resistant to confounding and hence the immortal time bias that arises from confounding by survival [8, 14]. This approach has been increasingly used to investigate the possibility of repurposing diabetic medications for prevention of other diseases [15, 16, 17], or possible drug targets for cancer prevention using a more agnostic approach by screening the entire druggable genome (e.g., proteomic Mendelian randomization [18]) although this requires downstream matching of relevant medications which may be challenging if the link of proteins with drug mechanism is not clearly understood. Prior Mendelian randomization studies of metformin on cancer yielded conflicting results, likely driven by methodological heterogeneity or limited sample size [19, 20, 21]. Furthermore, metformin's pleiotropic mechanisms suggested earlier studies might not have evaluated all relevant putative targets [22, 23]. To address these gaps, we conducted a drug‐target Mendelian randomization study assessing the association of putative targets of metformin with onset of five major cancers, including colorectal, breast, prostate, lung and endometrial cancer.

## 2 Methods

### 2.1 Study Design

A drug‐target Mendelian randomization design relies on the three instrumental variable assumptions (Figure S1) [24]. Firstly, the genetic variants must be functionally relevant to the perturbation of metformin targets (i.e., *cis*‐variants) (relevance). Secondly, there are no confounders of the genetic variant and the outcome (independence). Lastly, the effect of the genetic variants on the outcomes should only operate through the exposure (exclusion restriction). This study was reported following the Strengthening the Reporting of Observational Studies in Epidemiology Using Mendelian Randomization (STROBE‐MR) guideline (Supporting Information) [25].

### 2.2 Putative Targets of Metformin

Metformin primarily targets the liver and gut [23], exerting its pharmacological effects mainly through the activation of AMP‐activated protein kinase (AMPK), a key regulator of cellular energy homeostasis. Beyond its metabolic effects, metformin has also been shown to enhance anti‐cancer immunity by modulating the tumour immune microenvironment via both AMPK‐dependent and AMPK‐independent mechanisms [23]. AMPK functions as a heterotrimeric complex comprised of αβγ‐subunits, with human isoforms encoded by seven distinct genes: *PRKAA1* and *PRKAA2* (α1 and α2 subunits), *PRKAB1* and *PRKAB2* (β1 and β2 subunits), *PRKAG1*, *PRKAG2* and *PRKAG3* (γ1, γ2 and γ3 subunits). Although AMPK activation represents the canonical mechanism of action, the complete molecular pathways underlying metformin's effects remain incompletely characterised [22, 26]. To address this gap, we systematically identified additional putative targets from the literature and DrugBank (accessed May 13, 2025), a rigorously curated knowledge base for drug, drug‐target and related pharmaceutical information which is updated periodically [27]. Our final selection included 11 genes: the seven AMPK subunit genes *(PRKAA1, PRKAA2, PRKAB1, PRKAB2, PRKAG1, PRKAG2, PRKAG3)*, along with *ETFDH* (electron transfer flavoprotein dehydrogenase) [28], *GPD1* (glycerol‐3‐phosphate dehydrogenase 1) [29], *SLC47A1* (solute carrier family 47 member 1) [30] and *ACACB* (acetyl‐CoA carboxylase beta) [31], as detailed in Table S1.

### 2.3 Genetic Instruments for Putative Targets of Metformin

For the instrument selection, we first obtained tissue‐specific *cis*‐eQTL (*cis*‐acting expression quantitative trait loci within ±1 Mb of the transcription start site) for the 11 metformin target genes from GTEx v10 (*N* = 943, predominantly European ancestry) [32]. Given the sex‐specific nature of breast, prostate, and endometrial cancer, we obtained genetic associations with overall and sex‐specific HbA1c from the UK Biobank (*N* = 344 182, 185 022 women, 159 160 men, European ancestry). *Cis*‐eQTLs were retained as candidate instruments if they met the following criteria: (1) Nominal *p* < 0.05 for the associations of *cis*‐eQTL with HbA1c, and approximated F statistic (β_HbA1c_^2^/SE_HbA1c_^2^) > 10 to ensure robust instrument strength. (2) Independence: clumping (*r* ^2^ < 0.001 within 10 000 kb window) using the 1000 Genomes European reference panel to remove linkage disequilibrium (LD)‐correlated variants. (3) To validate biological relevance [33], we further assessed retained instruments against T2D, a well‐established metformin‐responsive outcome, using summary statistics from a genome‐wide association study (GWAS) (*N*_case_ = 242 283, *N*_control_ = 1 569 734) [34]. Only instruments showing a positive association with T2D at nominal significance (*p* < 0.05) were retained (Table S2). The full selection workflow is illustrated in Figure S2.

### 2.4 Exploratory Analyses

We also included two variants (rs11212617 [*ATM*] and rs8192675 [*SLC2A2*]) in the exploratory analyses as earlier studies suggested they modify metformin response [35, 36], and hence may also be possible targets of metformin aside from being an effect modifier. To ensure they are valid instruments, we assessed their association with HbA1c and T2D in the relevant summary statistics.

### 2.5 Genetic Instruments for HbA1c

To assess whether observed effects in our drug‐target Mendelian randomization design were through glycemic control pathways, we performed a complementary conventional Mendelian randomization design following our prior study [15]. Genetic instruments of HbA1c were also identified from the UK Biobank GWAS summary statistics based on three criteria: (1) Genome‐wide significant association with HbA1c (*p* < 5 × 10^−8^); (2) Independence from other selected variants (clumping *r* ^2^ < 0.001 within 10 000 kb window); and (3) Sufficient instrument strength (F statistic > 10). For analytical consistency within our drug‐target Mendelian randomization analysis and to account for potential sex differences, we used sex‐specific instruments when examining sex‐specific cancers (breast, prostate and endometrial), whereas we used both overall and sex‐specific instruments for non‐sex‐specific cancer outcomes.

### 2.6 Genetic Associations With Cancer Outcomes

The primary cancer outcomes were colorectal [37], breast (overall) [38], prostate [39], lung [40] and endometrial cancers [41]. To address tumour heterogeneity, secondary cancer outcomes included molecularly defined breast cancer subtypes [38]: luminal A‐like, luminal B‐like, luminal B/human epidermal growth factor receptor 2 (HER2)‐negative‐like, HER2‐enriched‐like, triple‐negative, *BRCA1* mutation carriers, oestrogen receptor (ER)‐positive‐like and ER‐negative‐like. Genetic associations with the outcomes were derived from the largest European ancestry restricted GWAS summary statistics obtained from the GWAS Catalogue [42], Table S3.

### 2.7 Statistical Analysis

We quantified instrument strength using the approximated F statistic (Beta_GX_/SE_GX_)^2^, with F statistic > 10 indicating minimal weak instrument bias [15]. For drug‐target Mendelian randomization analyses with limited instruments, we used the Wald ratio method. For conventional Mendelian randomization analyses of HbA1c on cancer outcomes, the main analysis was inverse‐variance weighted (IVW) regression with multiplicative random effects, which assumes balanced pleiotropy. We assessed potential horizontal pleiotropy through Cochran's Q test for heterogeneity and the MR‐Egger intercept. To assess robustness, we conducted sensitivity analyses using MR‐Egger (valid under the instrument strength independent of direct effect assumption), weighted median (requiring > 50% valid instruments) and weighted mode (plurality valid instruments) approaches [43]; consistent results across methods strengthen causal inference. To control for multiple testing, we applied a false discovery rate (FDR) correction (*α* = 0.05) separately for primary and secondary outcomes [44].

All analyses were performed using R version 4.4.1 with the ‘*TwoSampleMR*’ (version 0.6.15) and ‘*forestplot*’ (version 3.1.6) packages for statistical modelling and visualisation, respectively.

## 3 Results

Mendelian randomization analysis of 400 tissue‐specific *cis*‐eQTL and HbA1c associations across 11 metformin‐target protein‐encoding genes (*ETFDH, GPD1, PRKAA1, PRKAA2, PRKAB1, PRKAB2, PRKAG1, PRKAG2, PRKAG3, ACACB* and *SLC47A1*) revealed significant regulatory effects (*p* < 0.05), with 48 associations for overall HbA1c, 38 specific to women, and 30 specific to men (Tables S4–S6). After excluding weak instruments (F statistic < 10) and variants with discordant effects on T2D, two target‐specific instruments were identified: rs17237198 (*PRKAG1*, associated with both overall and sex‐specific HbA1c) and rs77023346 (*GPD1*, specific to overall HbA1c) (Table S7).

### 3.1 Genetically Predicted Target‐Specific Effects of Metformin on Cancer

**FIGURE 1:**
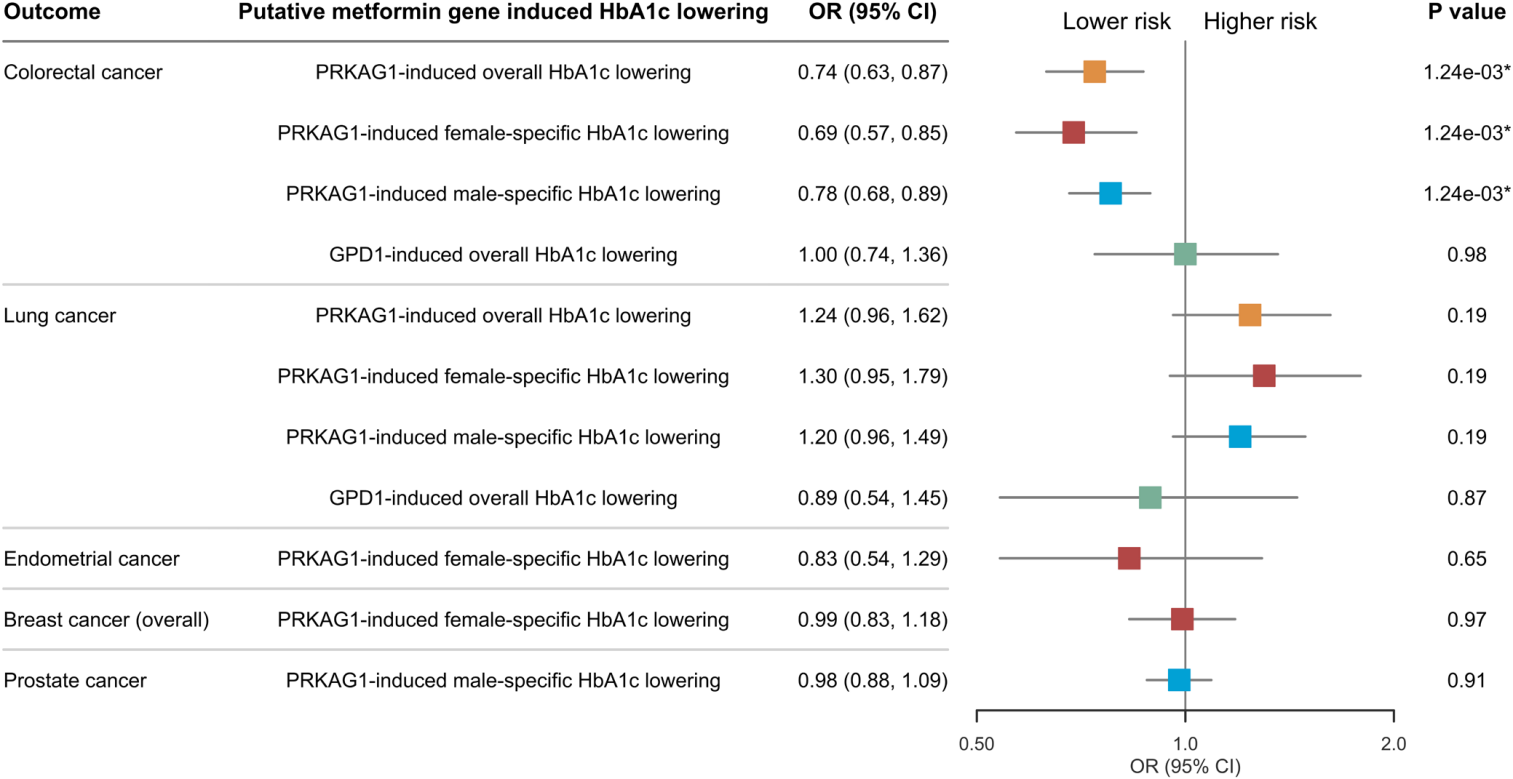
Genetically predicted target‐specific effects of metformin (per mmol/mol reduction in HbA1c) on primary cancer outcomes.

The drug‐target Mendelian randomization analysis, presented in Figure 1 and Table S8, evaluated the effects of metformin's putative targets (*PRKAG1* and *GPD1*) on the primary cancer outcomes. *PRKAG1*‐induced HbA1c lowering demonstrated a significant protective association with colorectal cancer risk (Wald ratio OR: 0.74 per mmol/mol reduction in overall HbA1c, 95% CI: 0.63–0.87; adjusted *p* = 0.001). This effect persisted sex‐specifically, with comparable risk reductions observed for male‐specific HbA1c lowering (OR: 0.78, 95% CI: 0.68–0.89; adjusted *p* = 0.001) and female‐specific HbA1c lowering (OR: 0.69, 95% CI: 0.57–0.85; adjusted *p* = 0.001). In contrast, *GPD1*‐induced HbA1c lowering showed no association with colorectal cancer (OR: 1.00, 95% CI: 0.74–1.36; adjusted *p* = 0.98). Neither target exhibited significant associations with lung cancer, or sex‐specific cancers (endometrial, breast and prostate cancer, all *p* > 0.05). Secondary outcomes analyses revealed no statistically significant associations after multiple testing correction (Figure S3).

### 3.2 Results From the Exploratory Analyses

rs11212617 near *ATM* had an F statistic far less than 10 (F statistic_max_ = 0.97) (Table S9), indicating that this was a weak instrument which could introduce bias and hence was not considered further. On the contrary, rs8192675 of *SLC2A2* was strongly associated with HbA1c and with low risk of weak instrument bias based on F statistics (F statistic_max_ = 124), with the expected association with T2D (Tables S9 and S10). Using this variant as the instrument, it was associated with lower risk of prostate cancer (OR: 0.89 per mmol/mol reduction in male‐specific HbA1c, 95% CI: 0.84–0.95; *p* = 0.004) (Table S11).

### 3.3 Genetically Predicted Effects of HbA1c on Cancer

**FIGURE 2:**
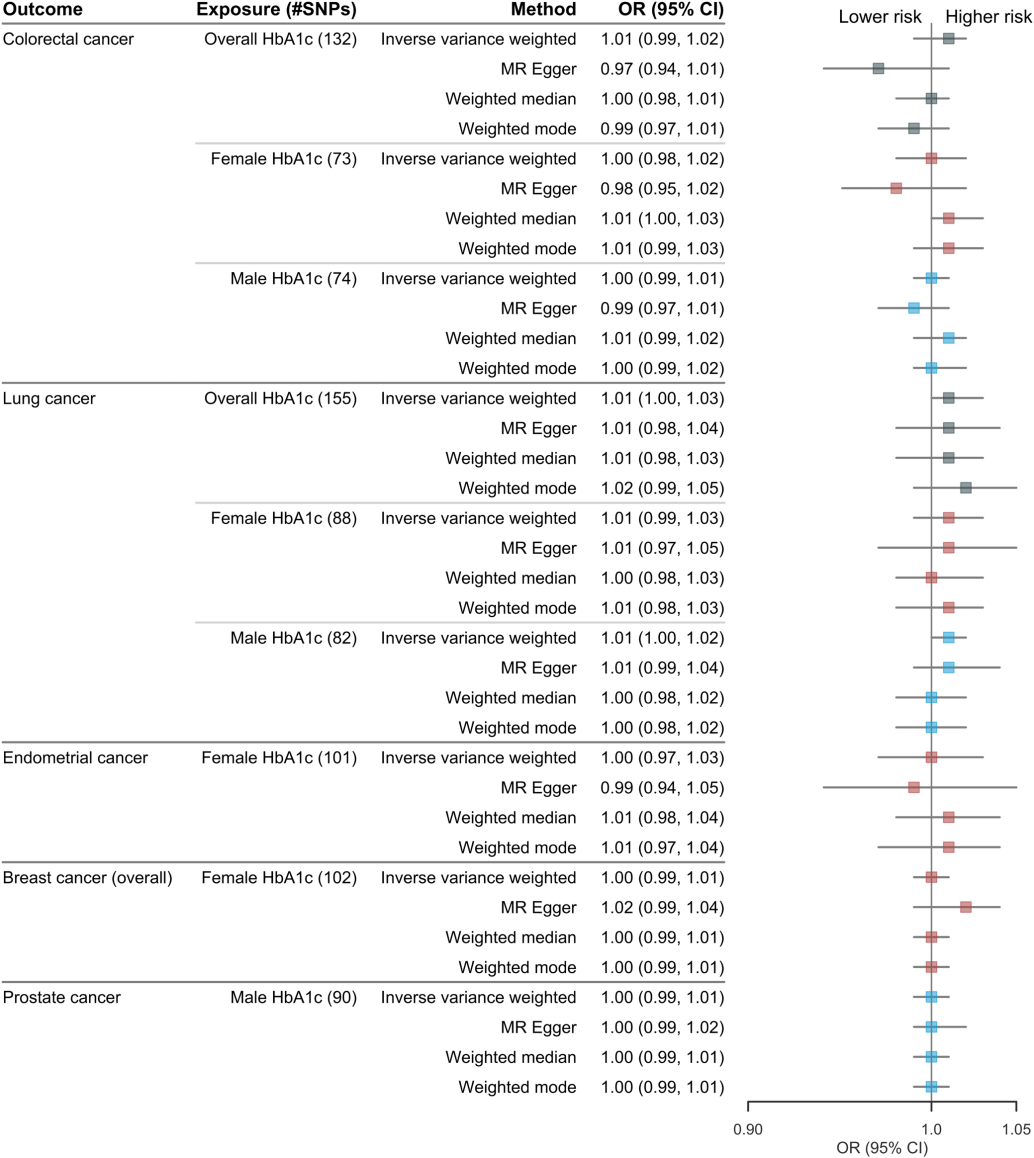
Associations of genetically predicted overall/sex‐specific HbA1c (per mmol/mol increase) with primary cancer outcomes.

We identified 185, 106 and 95 independent genetic instruments strongly associated with overall, female‐specific and male‐specific HbA1c (*p* < 5  ×  10^−8^), respectively (Table S12). All variants exhibited strong instrument strength (F statistics > 10), minimising concerns of weak instrument bias. As shown in Figure 2, genetically predicted HbA1c had no significant associations with any primary cancer outcomes, with consistent findings across sensitivity analyses. Although Cochran's Q test indicated substantial heterogeneity for all primary cancer outcomes, the MR‐Egger intercept test revealed no strong evidence for overall horizontal pleiotropy (Table S13). The null associations were also observed for breast cancer subtypes (Table S13 and Figure S4).

## 4 Discussion

This drug‐target Mendelian randomization study assessed the target‐specific effect of metformin on cancer prevention. Our study provides genetic evidence supporting the potential repositioning of metformin for colorectal cancer prevention, which is in line with earlier observational studies [45] and the American Gastroenterological Association clinical practice recommendation [46]. Notably, this effect appears specifically through its action on AMPKγ1 (*PRKAG1*) and is independent of glycemic control, which aligns with a prior Mendelian randomization study revealing no causal effect of glucose traits on colorectal cancer risk [47].

Existing pharmacoepidemiologic literature reports mixed associations of metformin with colorectal cancer risk, with some studies demonstrating lower colorectal cancer incidence [45, 48]. For example, a retrospective cohort study found an inverse association of prolonged metformin administration (≥ 5.0 years) with colorectal cancer risk, particularly for men, with minimised time‐related biases in the analyses [49]. However, another cohort study suggested null effects after accounting for immortal time bias through time‐dependent Cox, landmark, nested case–control and time‐fixed Cox analyses, although whether these methods can fully address such bias is unclear [50]. These discrepancies might be driven by a short follow‐up period (median follow‐up of 6.3 years), as colorectal carcinogenesis (e.g., adenoma to carcinoma progression) often spans 15+ years [50, 51]. Mechanistically, small RCTs support metformin's chemo‐preventive potential via LKB1/AMPK/mTOR pathway activation, evidenced by suppression of aberrant crypt foci (ACF, surrogate marker of colorectal cancer) and colon adenoma or polyp recurrence (signal for sporadic colorectal cancer) [52, 53, 54]. Although these trials were limited in scale, our study provides genetic evidence to independently validate metformin's candidacy for colorectal cancer prevention, warranting confirmation in large‐scale RCTs. The lack of associations of metformin targets with other cancers suggests tissue‐specific therapeutic effects.

### 4.1 Mechanisms of Association

The precise mechanism underlying metformin's effect on cancer remains incompletely understood, though AMPK activation represents the most well‐characterised pathway [55]. As a central regulator of cellular energy metabolism, AMPK dysregulation has been implicated in oncogenesis [56]. In many malignancies, the inactivation of the upstream kinase liver kinase B1 (LKB1) reduces threonine 172 (T172) phosphorylation of AMPK, diminishing its tumour‐suppressive activity and thereby promoting proliferation, angiogenesis, and metastasis [56, 57, 58]. Activated AMPK directly inhibits oncogenic mammalian target of rapamycin complex 1 (mTORC1) signalling—a key driver of protein synthesis, cell growth, proliferation, angiogenesis and promoting apoptosis—suggesting this axis as the primary mechanism of metformin's potential anti‐neoplastic effects [59, 60].

Our findings indicate that the mechanism via activated AMPK is specific to colorectal cancer, with emerging evidence implicating host‐gut microbiota interactions as a potential underlying mechanism [23], which is consistent with the role of AMPK in cancer in general being uncertain [61]. Metformin, through AMPK‐dependent pathways, directly modulates gut microbiota composition and the intestinal environment independently of its antidiabetic effects [23]. RCTs further support that metformin suppresses the recurrence of ACF and colon adenomas or polyps [52, 53]. Most clinical evidence on metformin's microbiota‐modulating effects is based on faecal microbiota, which primarily reflects the large intestine (consisting of cecum, colon, rectum and anus)—where metformin concentrations are highest [23]. These microbiota changes not only improve metabolic functions in the gut [62, 63] but also appear to contribute to metformin's specific anti‐cancer activity in colorectal tissue. In addition, somatic alterations in AMPK subunit genes may play a role in cancer‐specific outcomes. Copy number alterations in AMPK pathway genes have been identified across various cancers, with some genes amplified and others deleted depending on the tissue context [55, 64]. Whether these genetic expression changes affect downstream pathways and tumour suppression likely depends on tissue‐specific factors [64, 65], potentially explaining why metformin‐mediated AMPK activation prevents colorectal cancer but not the other cancers considered in our study.

### 4.2 Limitations

Despite leveraging a Mendelian randomization design to circumvent confounding, several limitations should be acknowledged. First, while we identified AMPKγ1 as a plausible target for colorectal cancer prevention, metformin's poly‐pharmacology—with multiple incompletely characterised mechanisms—suggests this likely represents only one component of its overall effect [66]. Each target or subunit may be differentially affected by metformin, and there may also be additional, yet unidentified targets involved. Furthermore, we do not know the proportion of effect mediated via these different pathways in contributing to the overall therapeutic effect of metformin. As such, we cannot accurately estimate the total therapeutic effect of metformin by using standard meta analytic approach [66]. Nevertheless, if all included targets show no appreciable effect on the outcomes, this can provide evidence against the presence of an effect of medication on the outcome. Second, the inability to perform colocalization given the lack of access to full summary statistics of genotype‐tissue expression leaves residual uncertainty about confounding by LD. Given the reliance of causal inference on a single variant (rs17237198 [*PRKAG1*]), it may tag regulatory elements affecting nearby genes, making it challenging to disentangle whether the observed associations are driven by the intended target or by correlated variants influencing other genes due to the absence of data to perform genetic colocalization. Despite these limitations, the consistent findings with the positive control outcome in the instrument selection process provide some reassurance regarding instrument validity. Third, Mendelian randomization cannot address all time‐related biases, particularly selection bias in cancer GWAS (e.g., survivor bias among older participants). The extent of selection bias depends on the age at disease onset and the proportion of deaths before study recruitment from the cancer of interest or a competing risk of that cancer, both of which vary across cancer types. The incidence of early‐onset colorectal cancer (< 50 years old) has been increasing [67]. Although this may reduce the likelihood of selection bias, we cannot rule out the possibility of selection bias as the GWAS of colorectal cancer included older participants and hence these participants had to survive multiple diseases to be included in this GWAS. More importantly, estimates for other later onset cancers, such as prostate cancer, may be biassed towards the null. Fourth, Mendelian randomization is better suited for testing causal hypotheses (e.g., the presence/absence of causation) rather than inferring therapeutic parameters (e.g., optimal dose/timing/duration), necessitating careful translation to RCT design [68] in the general population. Fifth, this study focused on metformin operating via one specific aspect of AMPK, which does not preclude metformin reducing cancer risk via other mechanisms, such as by reducing testosterone in women [69] and men [70], which could be relevant to the onset of breast, prostate and endometrial cancer. Sixth, our study primarily relied on DrugBank for drug target gene selection, which may not be exhaustive. Although we also included additional variants in *ATM* and *SLC2A2* as targets to improve coverage of instrument selection, earlier literature suggested their major role as effect modifiers instead of metformin targets and hence may invalidate the analyses. As such, findings from the exploratory analyses, should be interpreted in light of these possible issues. Seventh, stratified colorectal cancer subtypes (e.g., microsatellite instability [MSI] vs. microsatellite stability [MSS]) are currently unavailable in large‐scale GWAS summary statistics, limiting our ability to explore target‐specific effects of metformin in colorectal cancer subtypes. Furthermore, our study evaluates prevention rather than treatment effects—the latter requiring genetic data on disease progression, which is prone to selection bias [71]. Lastly, the predominantly European ancestry data used in this study underscores the need for cross‐population replication to evaluate generalizability.

## 5 Conclusions

This genetic association study found an inverse association of putative metformin target AMPKγ1 (*PRKAG1*) with colorectal cancer risk, likely independent of its glycemic properties. Our study provides genetic evidence supporting further investigation of metformin for colorectal cancer prevention; however, ethical considerations and other effects of metformin must be carefully addressed before proceeding with clinical trials, particularly in the general population.

## Author Contributions

S.L.A.Y. and S.L. designed the study. X.S. wrote the analysis plan and performed statistical analyses, with feedback from S.L.A.Y., S.L., and C.M.S. X.S. wrote the first draft of the manuscript with critical feedback and revisions from S.L.A.Y., S.L., C.M.S., I.C.K.W., E.Y.F.W., J.Z., and C.S.L.C. All authors contributed to reviewing and editing the manuscript and approved the final version to be published. X.S. is the guarantor of the work.

## Funding

This study is funded by Seed Fund for Basic Research for New Staff 2022/23 to S.L. (2201102266), The University of Hong Kong. The funder had no role in the design, analyses, interpretation of results or writing of the paper.

## Ethics Statement

This study only used publicly available summary statistics. Ethical approval was obtained for each of the original studies from which the data were sourced.

## Consent

Consent was obtained for each of the original studies from which the data were sourced.

## Conflicts of Interest

I.C.K.W. received research grants outside of submitted work from European Commission, Research Grants Council of the Hong Kong Special Administrative Region of the People’s Republic of China, Health and Medical Research Fund of the Government of the Hong Kong Special Administrative Region of the People’s Republic of China and Amgen; consulting fees from IQVIA and World Health Organization; served as a member of Pharmacy and Poisons Board of Hong Kong; was the founder and director of Therakind Limited (UK). He is the non‐executive director of Jacobson Pharma Corp. Ltd. in Hong Kong; and director of Advance Data Analytics for Medical Science (ADAMS) Limited (HK), Healthcare Innovation Technology Service (HITS) limited (UK) and OCUS Innovation Limited (HK, Ireland and UK). He received honorarium from Takeda as a speaker of continuous professional education study day. E.Y.F.W. has received research grants from the Health Bureau, the Hong Kong Research Grants Council, Narcotics Division, Security Bureau, Social Welfare Department, Labour and Welfare Bureau of the Government of the Hong Kong SAR and National Natural Science Foundation of China; serves on member of Core Team for Expert Group on Drug Registration of Pharmacy and Poisons Board, and is the director of Advance Data Analytics for Medical Science (ADAMS) Limited (HK). These are outside the submitted work. S.L.A.Y. received honorarium from Standard BioTools for presentation of proteomic studies irrelevant to this study. The authors declared no other Conflicts of Interest.

## Supporting information


**Figure S1:** dom70598‐sup‐0001‐Figures.pdf.

**Table S1:** dom70598‐sup‐0002‐Tables.xlsx.

**Data S1:** dom70598‐sup‐0003‐Supinfo.docx.

## Data Availability

Data collection were not performed as part of this study. All GWAS summary statistics used in this study are publicly available, with details provided in Table S3.
