# Supplementary material for: Evaluating the Impact of Putative Metformin Targets on Cancer Outcomes: A Drug‐Target Mendelian Randomization Study
Source: Diabetes Obes Metab. 2026 Feb 27;28(5):4091–9. doi: 10.1111/dom.70598 (PMC13071220; doi:10.1111/dom.70598)
Supplement: Supplementary file 1 — Figure S1: dom70598‐sup‐0001‐Figures.pdf. [file DOM-28-4091-s003.pdf]

Supplementary Figures

Supplementary Figure 1. Directed acyclic graph of the drug-target Mendelian randomization design

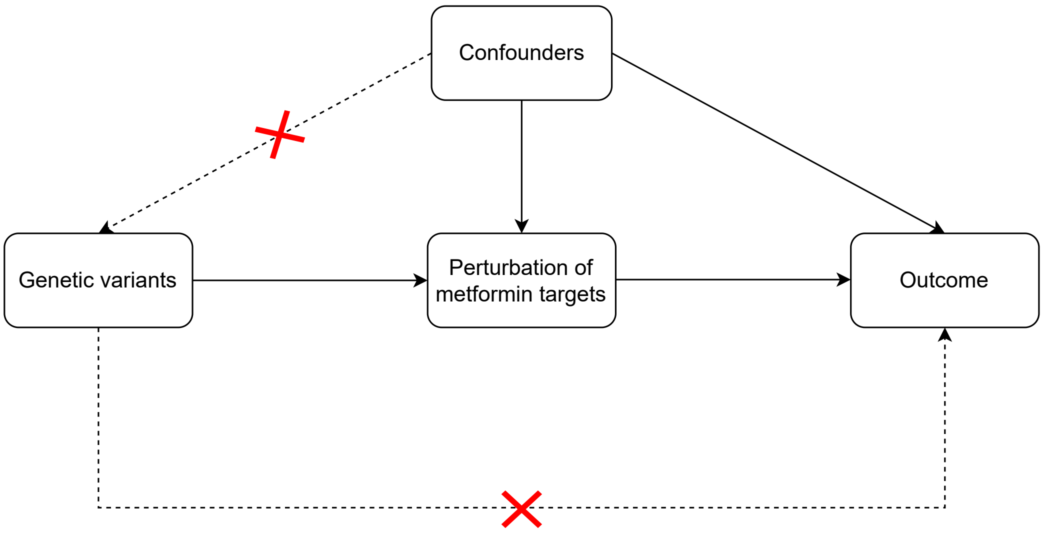

Supplementary Figure 2. Flowchart of genetic instruments selection for metformin's putative drug targets

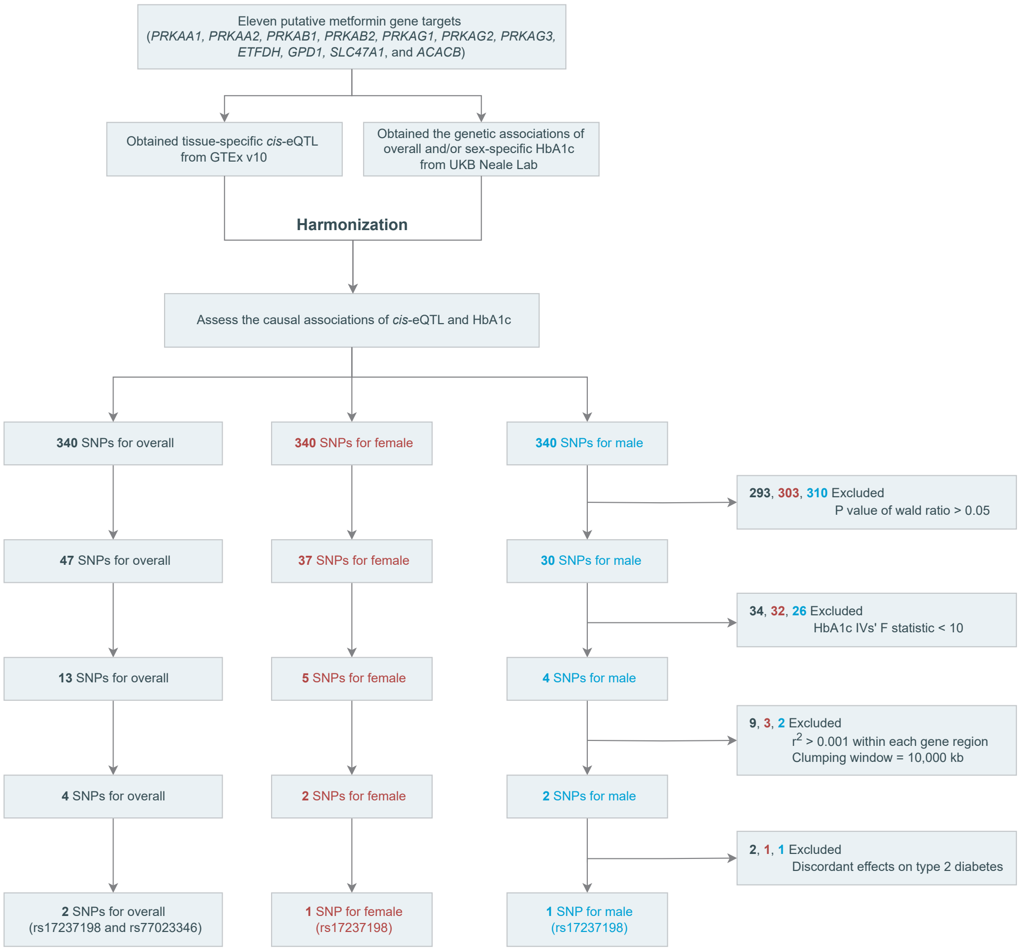

Cis-eQTL, *cis*-acting expression quantitative trait loci; UKB, UK Biobank; HbA1c, glycated hemoglobin; SNPs, single nucleotide polymorphisms; IVs, instrumental variables.

Supplementary Figure 3. Effect of metformin target *PRKAG1*-induced female-specific HbA1c lowering on breast cancer subtypes using drug-target Mendelian randomization

Outcome	OR (95% CI)	P value
Luminal A-like	0.94 (0.75, 1.19)	0.68
Luminal B-like	1.97 (1.17, 3.31)	0.13
Luminal B/HER2-negative-like	0.89 (0.58, 1.38)	0.68
HER2-enriched-like	0.57 (0.28, 1.18)	0.52
Triple-negative	0.81 (0.52, 1.26)	0.68
BRCA1 mutation carriers	0.74 (0.53, 1.04)	0.51
ER-positive-like	1.20 (0.84, 1.70)	0.68
ER-negative-like	0.89 (0.59, 1.36)	0.68

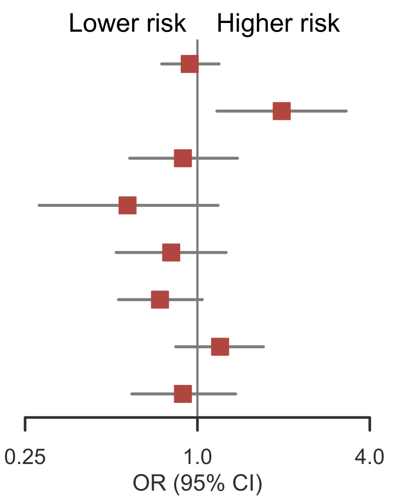

OR, odds ratio; CI, confidence interval; HER2, human epidermal growth factor receptor 2; ER, estrogen receptor.

Supplementary Figure 4. Associations of genetically predicted female HbA1c (per mmol/mol increase) with breast cancer subtypes

Outcome	Exposure (#SNPs)	Method	OR (95% CI)
Luminal A-like	Female HbA1c (101)	Inverse variance weighted	0.99 (0.97, 1.01)
		MR Egger	1.01 (0.98, 1.05)
		Weighted median	1.00 (0.98, 1.01)
		Weighted mode	0.99 (0.98, 1.01)
Luminal B-like	Female HbA1c (101)	Inverse variance weighted	1.01 (0.98, 1.03)
		MR Egger	1.01 (0.97, 1.05)
		Weighted median	1.00 (0.97, 1.04)
		Weighted mode	1.00 (0.97, 1.04)
Luminal B/HER2-negative-like	Female HbA1c (101)	Inverse variance weighted	0.99 (0.97, 1.01)
		MR Egger	0.99 (0.95, 1.03)
		Weighted median	1.00 (0.97, 1.03)
		Weighted mode	0.98 (0.95, 1.02)
HER2-enriched-like	Female HbA1c (101)	Inverse variance weighted	0.98 (0.95, 1.02)
		MR Egger	0.99 (0.93, 1.06)
		Weighted median	0.97 (0.92, 1.02)
		Weighted mode	0.98 (0.93, 1.03)
Triple-negative	Female HbA1c (101)	Inverse variance weighted	1.01 (0.99, 1.04)
		MR Egger	1.04 (0.99, 1.09)
		Weighted median	1.02 (0.99, 1.05)
		Weighted mode	1.02 (0.98, 1.05)
BRCA1 mutation carriers	Female HbA1c (102)	Inverse variance weighted	1.01 (0.99, 1.03)
		MR Egger	1.03 (0.99, 1.08)
		Weighted median	1.02 (0.99, 1.04)
		Weighted mode	1.00 (0.98, 1.03)
ER-positive-like	Female HbA1c (88)	Inverse variance weighted	0.97 (0.95, 1.00)
		MR Egger	1.00 (0.95, 1.05)
		Weighted median	0.97 (0.95, 1.00)
		Weighted mode	0.98 (0.95, 1.01)
ER-negative-like	Female HbA1c (88)	Inverse variance weighted	0.99 (0.97, 1.02)
		MR Egger	1.05 (1.00, 1.10)
		Weighted median	1.01 (0.98, 1.05)
		Weighted mode	1.04 (1.00, 1.07)

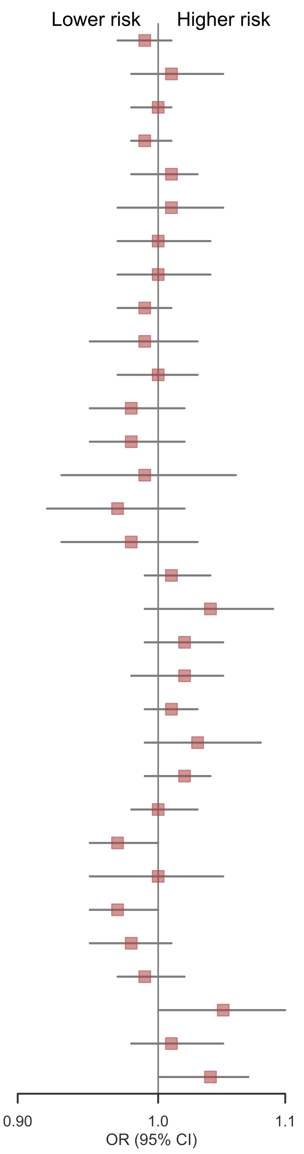

OR, odds ratio; CI, confidence interval; #SNPs, number of single nucleotide polymorphisms; MR, Mendelian randomization; HER2, human epidermal growth factor receptor 2; ER, estrogen receptor.
